# Navigating child and adolescent mental health in the digital age

**DOI:** 10.3389/fpsyt.2025.1514806

**Published:** 2025-05-23

**Authors:** Giorgia Di Iorio, Dario Esposito, Sara Romano, Fabiola Panvino, Benedetta Altomonte, Giulia Conte, Maria Pia Casini, Arianna Terrinoni, Dario Calderoni, Romana Andò, Walter Quattrociocchi, Benedetto Vitiello

**Affiliations:** ^1^ Department of Human Neuroscience, Unit of Child and Adolescent Neuropsychiatry, Sapienza University of Rome, Rome, Italy; ^2^ Department of Communication and Social Research, Sapienza University of Rome, Rome, Italy; ^3^ Department of Computer Science, Sapienza University of Rome, Rome, Italy; ^4^ Section of Child and Adolescent Neuropsychiatry, Department of Public Health and Pediatric Sciences, University of Turin, Turin, Italy

**Keywords:** social media, adolescents, mental health, screen time, well-being, depressive symptoms, body image

## Abstract

Since 2010, there has been a significant increase in mental health issues among children and adolescents, which coincides with the widespread use of social media (SM). While the exact relationship between SM use and psychiatric health remains unclear, growing evidence suggests that excessive screen time is linked to psychosocial symptoms. This article examines the importance of systematically assessing SM use as part of a comprehensive psychiatric evaluation for young individuals. Key factors to consider include the type, content, and purpose of SM use, as well as daily usage patterns and overall screen time. For instance, nighttime SM use can disrupt sleep and contribute to mental health issues. Additionally, while exposure to inappropriate content on SM may negatively affect mental health, positive online interactions can provide support and educational opportunities. Clinicians should also distinguish between different types of SM users—active creators, interactive participants, and passive consumers—since these engagement levels may influence mental health outcomes. Standardized tools for assessing SM use are recommended, though challenges arise due to the rapidly evolving digital landscape. Lastly, fostering open and non-judgmental communication with young patients about their digital habits is essential for understanding the role of SM in their lives and promoting healthier behaviors.

## 1 Introduction

In recent years, there has been a significant surge in mental health issues among children and adolescents, coinciding with the widespread adoption of social media (SM) since about 2010 (1–7). Almost one in five young people in Europe suffer from a mental disorder, with a prevalence rate of 15.5% (8). In particular, anxiety, obsessive-compulsive disorder, and depressive disorder show the highest prevalence (8). There is a growing consensus that the concurrent spread of SM among adolescents, with almost 95% reporting daily use (9), may influence youth mental health (1). SM has become ubiquitous, and the distinction between online and “real” life has blurred as these aspects have become intertwined. Although SM can provide benefits such as fostering peer interaction, rising emotional awareness, creating communities and promoting self-expression (10), the scientific literature seems to mainly highlight its negative effects, including increased anxiety (11), depression (12), psychological distress (13), eating disorders (14), sleep issues (15), and overall well-being (16). The pervasive and constant nature of SM could intensify certain vulnerabilities that are particularly pronounced during adolescence. While the exact role of SM in psychiatric health remains unclear, given the still limited evidence from adequate studies (17), there is a growing need for proactive approaches to assessing SM use in children and adolescents. As a guide for parents, the principal international health regulatory bodies (e.g., World Health Organization [WHO]) in 2019 recommended no screen time for children up to 1 year of age, no more than 1 hour for 2-year-olds, and the least screen time possible preferred for children aged 3 to 5 years. These limitations are also recommended to be extended to older children and adolescents through parental management (18). However, these recommendations risk being questioned, especially when considering adolescents, who often show poorly controlled use of the internet and SM, and for whom parents lack clear guidelines on how to moderate usage (9, 19). Additionally, it is clear that screen-time limits alone may be excessively rigid and difficult to implement in everyday family life. There are sufficient reasons to recommend that a systematic assessment of SM use be part of a comprehensive psychiatric evaluation of children and adolescents.

Based on existing literature and clinical experience, we examine the key elements to consider in such an assessment, including SM type, content, purpose, extent, and daily patterns of use (**Table 1**).

**Table 1:** A structured assessment designed to help clinicians in gathering essential information regarding the relationship between social media (SM) use and the mental health of children and adolescents.

Assessing the Use of Social Media by Children and Adolescents	
**Screen Time**	*How much time do you* sp*end on screens each day? How much of that is dedicated to social media?* Inquire about daily/weekly screen usage, including smartphones, tablets, TV and computers. If possible, assess with the patient the SM app time usage directly through the smartphone time monitoring features.
**Usage Patterns**	*When do you* usually *use social media? Is it more during the day or at night?* Explore the timing of SM engagement, considering potential associations with sleep disturbances (e.g., risk of “vamping”).
**Type of Social Media**	*Which social media platforms do you use? For example, TikTok, Instagram, Snapchat…?* Identify specific platforms to understand the diversity of experiences, including image-based or text-based content.
**Content Preferences**	*What kind of things do you like to see or share on social media?* Explore the content they engage with, distinguishing between short-form videos, images, or text-based information.
**Exposure to Mental Health-Related Content**	*Have you come across content related to mental health issues (like self-harm, eating disorders, or tics) on social media? How do you feel after seeing such content?* Discuss the patient’s exposure to content related to their mental health issues. Explore potential risks such as reinforcement of negative behaviors, suggestion, and misinformation, while also considering the possible benefits, including identity exploration, community support, and access to accurate psychoeducation.
**User Activity**	*Do you actively post and create content on social media, do you interact with other users’ contents, or do you just observe?* Understand their level of engagement, distinguishing between active content creation, interactive behaviors, and passive observation.
**Social Connections**	*Do you connect with friends on social media, or do you mainly follow influencers or strangers?* Explore the nature of their social connections, differentiating between personal connections and following online celebrities.
**Psychological Dimensions**	*Do you ever compare yourself to others on social media? How do you feel if you miss out on something? Do you ever post vague or ambiguous messages on social media?* Assess psychological aspects, including social comparison, fear of missing out (FOMO), fear of being ignored (ghosting), and vaguebooking (i.e., posting unclear messages that invite speculation about their emotional state). Consider using questionnaires to explore several different psychological dimensions.

## 2 Discussion

### 2.1 Temporal dynamics of SM Use

Examining the temporal aspects of SM use is crucial for a comprehensive understanding of its impact on mental health. This involves assessing total screen time across smartphones, tablets, and computers, though excessive use is just one of several factors to consider. For instance, daytime engagement might differ significantly from nighttime interactions, influencing the individual’s social dynamics and emotional experiences. It is essential to explore whether adolescents predominantly use SM during solitary hours or in the company of others, as this can shed light on the role of SM in substituting or complementing real-life social connections. On the other hand, SM use may be linked to poor sleep quality in adolescents. Behaviors such as “vamping,” characterized by compulsive nighttime use of digital devices, are associated with individuals who become “hooked” on these activities, potentially disrupting sleep-wake rhythms, leading to frequent awakenings and daytime sleepiness (20). In the literature, there is strong evidence linking shorter sleep duration, daily dysfunction, and longer sleep latency with general screen time (21). The effects of screen time on sleep quality could be mediated by direct mechanisms as well as psychosocial factors. Among the direct mechanisms, we find blue light exposure, known to be an activating factor that alters melatonin secretion and causes delays in sleep onset (22), and unique features of SM: constant notifications and alerts. These characteristics disrupt adolescents’ sleep-wake rhythm, especially considering that approximately 86% of adolescents sleep with their phone in the bedroom or under their pillow (15). The psychosocial mechanisms, on the other hand, arise from the pressure to be available 24/7. Adolescents report anxiety when their access to SM is restricted during the night, and they cannot reply to a message immediately (23, 24). It is possible that young people have difficulty relaxing at bedtime due to the fear of missing out (FOMO) on their friends’ activities (24). Additionally, adolescents may have social commitments that overflow into the night or engage in rumination about issues with others that can disrupt their sleep (25). The nature of SM, coupled with the allure of constant connectivity, may lead to prolonged device use at the expense of adequate sleep, thereby creating a cyclical relationship between excessive SM use, sleep disturbances, and deteriorating mental health.

### 2.2 Content and purpose of SM use

The impact of SM content on mental health is crucial for clinicians to address. Conditions like dissociative identity disorder (26), tic-like disorders (27), eating disorders (28), and non-suicidal self-injury (26) have been increasingly associated with excessive or inappropriate SM exposure (29). Clinicians must inquire not only about the platforms used but also the nature of the content shared and searched. SM may serve as a source of support and education but also a potential breeding ground for maladaptive behaviors, including self-diagnosis and contagion. Furthermore, delineating the predominant types of SM content is crucial: those centered on images and videos (referred to as “highly visual” SM [HVSM], such as Instagram or TikTok) show notable differences from text-based platforms (e.g., X - previously known as Twitter - or WhatsApp). In particular, HVSM encourage a focus on physical appearance, and by the ability to access users’ photos and videos at any moment of the day, they lead to constant comparisons with peers (30). In some studies, the relationship between trends in posting modified selfies on HVSM (i.e., using filters or apps to manipulate photos) and mental health issues was analyzed. In young people who invest more time in taking and posting selfies, lower body satisfaction and an increased prevalence of eating disorders were found (31). Additionally, other studies have linked the use of HVSM to negative effects on self-esteem and body image concerns. Marengo and colleagues found that adolescents reporting higher use of HVSM expressed higher dissatisfaction with their bodies and reported greater levels of emotional symptoms; these effects were stronger than in adolescents using text-based platforms (32). Continuous exposure to unrealistic or unattainable physical beauty standards on these platforms can exacerbate feelings of inadequacy and contribute to the development of internalizing symptoms. However, in addition to these negative aspects for adolescents’ mental health, the widespread use of devices with internet access has made them a fundamental means for young people to develop their interests, build their sense of identity, and strengthen social networks outside the family (33). Positive implications have also been observed in younger children, particularly with respect to the use of stimulating and customizable learning platforms, which can be adapted to the needs of children with special educational needs. Because of this controversial nature of the effects of SM on young people, questioning them about how they use it and what content they expose themselves to is of paramount importance in the clinical practice of mental health professionals.

### 2.3 Addressing exposure to mental health-related content on social media

In addition to understanding the general patterns of SM use, it is critical for clinicians to discuss with patients their exposure to content related to the specific mental health issues they are facing. SM can be a double-edged sword in this context. On one hand, exposure to content about mental health problems like self-harm, eating disorders, or tic-like behaviors can reinforce negative patterns, increase suggestibility, and spread misinformation. For example, echo chambers or viral trends might normalize harmful habits or present them as coping mechanisms, potentially exacerbating the patient’s condition. On the other hand, exposure to mental health-related content can also play a positive role. In particular, the diffusion of content created by mental health professionals about psychological or psychiatric issues on SM, enables greater awareness among both young and adult users. This type of communication about mental health seems more accessible for adolescents looking for quick information on the internet (34). Additionally, the presence of profiles recounting their experience with mental health issues could be useful in the patient’s journey by providing identity exploration and community support. Engaging patients in discussions about the type of mental health-related content they encounter on SM allows clinicians to address the potential risks while also recognizing and encouraging the benefits, such as connecting with supportive communities or accessing reliable information.

### 2.4 Active vs. passive SM users

Understanding the nature of SM engagement is vital. Clinicians should discern whether young individuals are mainly content creators (i.e., contributory users), interactors (commenting and liking contents produced by others), or passive users (who merely consume information) (35). This distinction can provide valuable insights into the individual’s relationship with SM and its potential impact on their mental health. Analyzing the negative impact of SM usage type on mental health, a greater number of hours spent on SM (passive use) predict lower life satisfaction and have an inverse association with positive emotions while the number of contents shared (active use) predict higher life satisfaction (36). In particular, it should be inquired about the level of mental effort put in SM use and the perceived gratification resulting: problematic SM use and consequential mental distress could be associated with a high level of effort from which results low gratification (37).

### 2.5 Standardized questionnaires and assessments

Utilizing standardized questionnaires tailored for children and adolescents might be extremely useful in evaluating problematic SM use and its associated conditions. Nevertheless, it is imperative to acknowledge that the standardization and validation of such assessment tools demand considerable time and effort. Conversely, the landscape of SM evolves rapidly, driven by market demands. In this dual-speed process, there is a risk of rapid obsolescence of available tests, potentially failing to capture the most pertinent phenomena, especially considering the variety of SM platforms in existence. Most of the questionnaires adopted in clinical practice do not differentiate between various types of platforms, often failing to capture the profound difference in the nature of different SM and consequently of their effect on young people’s mental health. Moreover, most SM assessments focus primarily on the addiction model to address problematic SM use. This model, which has been widely adopted over the years, should be coupled with an analysis of other factors that contribute to distress and may not be in line with classic models of addiction (e.g., fear of missing out or fear of ghosting) (38). Additionally, placing excessive emphasis on screen time alone may overlook other facets of problematic SM use.

### 2.6 Direct communication with young patients

Engaging in open and non-judgmental conversations about SM use with young patients is pivotal. Showing curiosity and interest in their digital lives can foster trust and provide a comprehensive understanding of SM’s role in their overall well-being. In clinical practice, mental health professionals often face challenges when investigating the use of digital platforms by their patients. Similarly, young people may often report feeling judged or not understood when addressing these topics because of their clinician’s unfamiliarity in dealing with SM use. A dual need in the field of SM and mental health is evident. On the one hand, professionals can benefit from guidance to improve their ability to investigate SM use in their patients. On the other hand, young people want to feel comfortable talking about their online activities during consultations with mental health professionals, both to address challenges and to take advantage of the opportunity to discuss their experiences, seek support and develop coping strategies related to web safety (39).

In light of what has been discussed, we propose a structured assessment aimed at exhaustively investigating the use of SM and their potential effect on mental health in children and adolescents.

## 3 Conclusions

As the landscape of child and adolescent mental health evolves in tandem with the digital age, clinicians must adapt their approaches to address the challenges posed by SM. Given that SM use is now a relevant part of the daily lives of young people, it is critical for healthcare professionals to gain a comprehensive understanding of how digital platforms shape adolescent behavior and well-being. This requires more than just a cursory inquiry into screen time; clinicians should integrate structured SM assessments into their routine assessments, carefully exploring not only the frequency and duration of use but also the content, interactions, and emotional responses linked to SM engagement. While the surge in mental health issues among young individuals, such as anxiety, depression, and sleep disturbances, appears to parallel the widespread adoption of SM, it is essential to recognize that SM are not inherently detrimental. Moreover, the type of engagement with SM, along with other contributing factors such as pre-existing vulnerabilities and environmental influences, can either mitigate or exacerbate its potential risks on adolescents’ health. Clinicians must be prepared to shed light on both the risks and benefits associated with SM use, helping their young patients navigate the digital world with a critical and informed perspective. A proactive approach enables clinicians to engage in open, non-judgmental dialogues with their young patients, fostering trust and mutual understanding. As we move forward, it is imperative to advocate for the development of standardized assessment tools that adapt to the ever-changing landscape of SM use in adolescence. By embracing a holistic understanding of SM use, clinicians can harness the positive potential of digital connectivity while safeguarding the mental well-being of young individuals in the digital age.

## Data Availability

The original contributions presented in the study are included in the article/Supplementary Material. Further inquiries can be directed to the corresponding author.
